# Use of Nutraceuticals for Prevention and Treatment of Cancer

**Published:** 2013

**Authors:** Ahmad Salami, Enayatollah Seydi, Jalal Pourahmad

**Affiliations:** Department of Pharmacology and Toxicology, School of Pharmacy, Shahid Beheshti University of Medical Sciences Tehran, Iran.

Nutraceuticals, mostly phytochemicals derived from dietary or medicinal plants such as soya bean, garlic, ginger, tea as well as propolis, honey and others, may have chemopreventive activities, as already suggested by epidemiologic and animal model studies. Their ability to reduce cancer incidence in these studies is likely related to apoptosis. The potential of using nutraceuticals as chemopreventive reagents has prompted a surge of in-vitro studied on of their biological effects in cultured human cells. Chemoprevention is the use of small molecules, including dietary or herbal chemicals, to prevent cancers, as opposed to chemotherapeutics, where chemicals, mostly synthetic, are used to remove or alleviate cancer symptoms. The concept of chemoprevention, although prevalent in the East for thousands of years, has not gained scientific recognition in the West until recently. Large scale clinical studies have demonstrated the efficacy of using tamoxifen, raloxifene, both estrogen receptor antagonists, and fenretinide, a synthetic retinoid, in protecting women from breast cancer.

The report by the Chemoprevention Working Group to the American Association for Cancer Research was a watershed that signaled the acceptance of chemoprevention as a viable alternative means in cancer control. Therefore, it is of interest to explore the possibility of using phytochemicals or other dietary chemicals as chemopreventive agents. Beside, the study of the biological effects of these phytochemicals at cellular level provides the molecular basis for their anti-tumor function and helps to establish the platform for generating more potent chemopreventive and even chemotherapeutic agents. Apoptosis is involved in a whole array of normal physiological processes, including immune defense, tissue homeostasis and development, and any tilt of the balance between life and death within an organism which can lead to absence or enhancement of diseases. Thus, the loss of essential cells of post-mitotic tissues due to enhanced cell death may play an important role in a number of functional deficiencies and degenerative diseases such as Alzheimer’s disease, Parkinson’s disease, Huntington’s disease, multiple sclerosis, myocardial infarction, arteriosclerosis, chronic inflammation, rheumatoid arthritis, sterility, or cataract. However, apoptosis can be considered as a proactive selfdefense mechanism of a living organism to weed out dysfunctional cells such as the precursors of metastatic cancer cells, without creating secondary oxidative stress due to inflammation. Indeed, defect in apoptosis mechanism is recognized as an important cause of carcinogenesis. A dysregulation of proliferation alone is not sufficient for cancer formation; a suppression of apoptotic signaling is needed. Cancer cells acquire resistance to apoptosis by overexpression of antiapoptotic proteins (Bcl-2,IAPs, and FLIP) and/or by the downregulation or mutation of proapoptotic proteins (Bax, Apaf-1, caspase-8, and death receptors). Overexpression of antiapoptotic Bcl-2 and Bcl-xL probably occurs in more than 50% of all cancers. Flavonoids are a group of more than 4000 polyphenolic compounds that occur naturally in foods of plant origin. These compounds possess a common phenylbenzopyrone structure, and they are categorized according to the saturation level and opening of the central pyran ring, mainly into flavones, flavonols, isoflavones, flavonols, flavanone, and flavanonols. Among them, tea polyphenols, quercetin, and genistein have been widely studied for their potential chemopreventive applications. Some flavonoids were first shown to be apoptotic in human lymphoid leukemic cells and human carcinoma cells. Similar observations have since been extended to lung tumor cell lines, colon cancer cells, breast cancer cells, prostate cancer cells, stomach cancer cells, brain tumor cells, head and neck squamous carcinoma, and cervical cancer cells. Recent epidemiologic studies have shown good correlation between dietary intake of carotenoids and reduced risk of cancer and cardiovascular diseases.Tomato is rich in various carotenoids. Lycopene is the precursor of *β*-carotene in tomato, which accumulates after the lycopene cyclase gene is downregulated during ripening. Lycopene and *β*-carotene can induce apoptosis in prostate cancer cells and malignant lymphoblast cells. Caffeic acid phenethyl ester, an active phenolic component extracted from honey bee propolis, blocks tumorigenesis in a two stage model of mouse skin cancer. Caffeic acid phenethyl ester has been reported induce apoptosis in HL-60 leukemic cells and mouse epidermal JB6 Cl 41 cells. Curcumin induces apoptosis in colon carcinoma cells, leukemic cells, prostate cancer cells, melanoma cells, and breast cancer cells. The use of garlic as anticancer agent has long been established. The allylsulfur compounds derived from garlic have significant anti-proliferate activity against human cancers. Diallylsulfide and diallyldisulfide induce apoptosis in non-small cell lung cancer cells and in prostate cancer and breast cancer cells. With our increased understanding of the chemistry and biology of nutraceuticals, the nutraceutical research will shift more into the area of chemoprevention. It is suggested that the following considerations should be taken on the future use of nutraceuticals for disease prevention: 

1) synthesis of analogues; to further increase the efficacy of a promising nutraceutical. One can use it as a chemical template for combinatorial synthesis. 2) identification of molecular targets. With the molecular targets of nutraceuticals being known, it may be possible to develop more refined chemicals that specifically target those commonly shared sites. 3) synergistic effect: with the understanding of the molecular action of each nutraceutical, one can test possible synergistic effects on chemo-prevention by using two or more nutraceuticals or derivatives.

*Jalal Pourahmad is currently working as a professor at the Department of Pharmacology and Toxicology, School of Pharmacy, Shahid Beheshti University of Medical Sciences, Tehran, Iran. He could be reached at the following e-mail address: j.pourahmadjaktaji@utoronto.ca

